# Out-of-Hospital Administration of Medication without Prescription and Associated Factors among Preschool Children

**DOI:** 10.1155/2017/5242048

**Published:** 2017-10-10

**Authors:** Fotini Andritsou, Vassiliki Benetou, Koralia A. Michail, Nikolaos Pantazis, Ioanna D. Pavlopoulou

**Affiliations:** ^1^Pediatric Research Laboratory, Faculty of Nursing, National and Kapodistrian University of Athens, Athens, Greece; ^2^Department of Hygiene, Epidemiology and Medical Statistics, Faculty of Medicine, National and Kapodistrian University of Athens, Athens, Greece

## Abstract

The increasing trend of administering nonprescribed medicines in children is a significant public health issue. The aim of the present study was to assess the use of medication without a prescription (MWP), including both nonprescribed medication (NPM) and prescription-only medication (POM), and identify associated factors, among preschoolers in Athens, Greece. A predesigned questionnaire was distributed to parents from May through June 2011. Multivariable binary logistic regression analysis models were used to assess associations of interest. Results showed that 95.1% of parents reported administering at least one MWP, during the previous 12 months. Antipyretics (91%) were the most commonly NPM and bronchodilators (24.8%) and antibiotics (16.4%) the most common POM dispensed. Child's increased age group, lack of parental information, higher paternal education, and mother's foreign nationality were associated with increased antipyretic use (*p* < 0.05), while father's foreign nationality and parental age were positive predictors of antibiotic administration (*p* < 0.05). The likelihood of consuming antipyretics and antibiotics significantly increased when information was provided by a pharmacist (*p* = 0.017 and *p* = 0.054, resp.). Conclusively, most parents have administered at least one MWP, including antibiotics, to address symptoms of common childhood diseases, highlighting the need of information campaigns concerning the consequences of their improper use.

## 1. Introduction

A trend towards increased use of over-the-counter medicines, namely, those drugs that can be administered without a prescription (nonprescribed medication, NPM), has been observed in several developed countries, even among younger children [1, 2]. These include analgesics-antipyretics, cough and cold medicines, decongestants, and antihistamines, as well as various dermatological preparations, laxatives, and antiseptics [3, 4].

Studies have shown that increased consumption of NPM is associated with an increased rate of side effects, especially when administered without guidance from a health professional [1, 2, 5]. Recent estimates describe that 8% of pediatric emergency department visits are related to medication use, and 65% of them are deemed preventable [5]. According to information provided by the Poison Control Centre of the United States, over 200,000 cases of medication-related errors have been recorded since 2003 in the community, with 30% involving children [6]. Additional reports state that 23–73% of preschool children have taken NPM in the past [1, 2, 7]. Although there is plenty of information on the consumption of NPM among children in several countries, evidence regarding the reasons or indications which prompt parents to their use, as well as other associated factors, is limited [7–9].

In Greece, data on consumption of NPM in children lacks despite the fact that these were, until recently, provided only by a pharmacist with or without a doctor's prescription. Moreover, illicit dispensing of prescription-only medicines (POM), including antibiotics, at local pharmacies without a medical prescription, remains a standard practice [10, 11].

The aim of the present study was to evaluate the frequency of administration of medicines without the use of a prescription (MWP) for both NPM and drugs that require a prescription but are paradoxically dispensed without it (POM), in a sample of preschool children in the Municipality of Athens. A secondary objective was to investigate the factors associated with the administration of two representative categories—analgesics-antipyretics and antibiotics—in the above population.

## 2. Materials and Methods

### 2.1. Population

A cross-sectional study was conducted at the public kindergartens of the Municipality of Athens from May through June 2011. The above area consists of 98 nursery schools with a total of 5,401 registered pupils of middle and low-income families. Parents of children less than six years of age were eligible for participation.

### 2.2. Instrumentation

A modified questionnaire, comprising 11 sections, based on previously published surveys with the approved consent of the authors, was used for data collection [1, 2, 7–9]. Its format was finalized after a pilot test in a small number of parents that were excluded from the final study. All school teachers were informed through interactive sessions organized by the investigators. Parents/guardians were informed through both faculty and an explanatory letter and were asked to complete the questionnaires that were collected three weeks later. Consent was implied by returning of a completed questionnaire. The study protocol was approved by the review board of the Municipal Nursery of Athens.

### 2.3. Statistical Analysis

Data analysis was performed with the use of the Statistical Package for Social Sciences (SPSS) Ver.17. The mean values and standard deviations or the median values and interquartile range (IQR) were calculated for continuous variables, and frequencies and percentages were calculated for categorical and arranged variables.

Multiple variable analysis was used to draw quantitative conclusions for the 13 categories of MWP (NPMs and POM) in relation to the symptoms for which they were administered by the corresponding number of parents. Symptoms reported by the parents were grouped to the most frequent and were coded with the use of multiple response sets. Questionnaires in which parents did not state the symptoms, for which they administered a particular MWP, were excluded. Multivariable Binary Logistic regression was used to investigate the association of each of the 13 MWP categories with demographics, the way medicines are prescribed, the sources sought for information regarding their administration, and the reasons that motivated or discouraged parents to buy and use them, at the significance level 5% or 10% in some cases. The results were presented in Odds Ratios (OR) together with the corresponding *p* values and 95% (CI) Confidence Intervals. The variables that were selected to be used in the final model of multiple logistic regression analysis were based on the international literature and at statistically significant results of the univariate analysis (univariable binary logistic regression) and *χ*^2^ test. The most frequent category in each variable/factor was selected as reference category (OR = 1) in the categorical variables multifactorial models, whereas the assessment of each associated factor was weighted for all other factors in the model.

## 3. Results

A total of 4,608 questionnaires were distributed to 87 kindergartens of the Municipality of Athens, and 1,985 were returned (participation rate: 43.1%). The mean age of parents was 36.6 ± 5.2 years while the majority of respondents were mothers (79%). A percentage of 88% of parents had positive marital status with 74.3% of both parents being Greeks. Most of them were high school graduates (46.3% and 44.7% for paternal and maternal education, resp.) while in 29% at least one had higher education. Among children that had received MWP within the 12-month period before the study, 57.2% were firstborns, whereas 65.5% had siblings. Among participating parents, 95.1% stated administering at least one of the 13 categories of medicines included in the questionnaire, without a medical prescription. Of these 13 categories, antipyretics-analgesics (91%) were administered more frequently, followed by dermatological preparations (37.3%) and cough and cold medicines (34.4%). Among POM, bronchodilators (24.8%) were the preferred category, followed by antibiotics (16.4%).

The majority of parents admitted that fever was the main reason for using antipyretics- analgesics NPM (78%). Acetaminophen was the preferred antipyretic (57.2%), followed by mefenamic acid (35.1%) (Table 1). The most common use of antibiotics was due to upper respiratory infection (45.5%), with amoxicillin being the formulation of choice (41.4%, Table 2).

### 3.1. Factors Associated with the Administration of Antipyretics-Analgesics

According to the results of multiple logistic regression (Table 3), children belonging to the age group of 4–6 years were more likely to receive antipyretics-analgesic drugs compared to those aged 2–4 and those older than 6 years [(OR = 0.61, *p* = 0.013) and (OR = 0.07, *p* = 0.004), resp.]. Fathers with postgraduate education were 64% less likely (OR = 0.36, *p* = 0.002) to administer antipyretics-analgesics, compared to secondary school graduates, whereas mothers with Greek nationality were 25% less likely to use the above medication category when compared to mothers of foreign nationality (OR = 1.75, *p* = 0.038).

Parents not familiar with the term “medicines without prescription” appeared to have lower possibilities (OR = 0.26, *p* ≤ 0.001) of administering antipyretic-analgesics, compared to those who knew the meaning of the term. Notably, those who did not respond to the above query were nearly three times more likely (OR = 3.27, *p* < 0.001) to administer the medicines mentioned above.

The rapid and timely communication between parents and the pediatrician, in less than one day, seemed to reduce the likelihood of administering antipyretic-analgesics by 62% (OR = 0.38, *p* < 0.001) compared to the delayed one. Reduced risk of delivering antipyretics-analgesics was observed among parents that were uncertain and lacked appropriate information regarding the administered NPM (OR = 0.49, *p* = 0.009), as well as among those who did not provide any answer to the query on being aware of the active ingredients of the NPM they wished to administer (OR = 0.25, *p* = 0.003).

Parents who did not receive information from various sources, such as the pharmacist, the Internet, relatives, or magazines, appeared to have a reduced likelihood of administering antipyretic-analgesics by 53% (OR = 0.47, *p* = 0.030). However, increased likelihood of administration of the medicines above was observed when information was obtained by the pharmacist (OR = 1.77, *p* = 0.017) and by relatives (OR = 7.47, *p* = 0.030). In contrast, those informed by television and the Internet had a reduced likelihood of administration by 96% (OR = 0.04, *p* < 0.001) and 62% (OR = 0.38, *p* = 0.016), respectively.

### 3.2. Factors Associated with the Administration of Antibiotics

Administration of antibiotics without prescription was 53% more likely when the father was the responding person (OR = 1.53%, *p* = 0.012), increased by 2.2 times if the father was of foreign nationality and by 2.6 times (OR = 2.62, *p* = 0.009) if the question regarding citizenship was not completed.

Those who did not agree with the practice of buying medicines with the use of a previous prescription in order to manage a similar health problem of their child, as well as those who did not agree that a familiar brand name of a drug should drive to its purchase without a prescription, were less likely to administer antibiotics by 39% and 40%, respectively [(OR = 0.61, *p* = 0.001) and (OR = 0.60, *p* = 0.001)]. Finally, those disagreeing with the view that the administration of medicines should be preceded by a visit to the pediatrician were 2.8 times more probable to administer antibiotics without prescription (OR = 2.81, *p* < 0.001).

Similarly to the analgesic-antipyretic category, the provision of relevant information by the pharmacist appeared to increase the likelihood of antibiotic administration without a prescription by 31% (OR = 1.31, *p* = 0.054) (Table 4).

## 4. Discussion

This is the first study to provide information concerning the out-of-hospital consumption of MWP, including NPM and POM, as well as the factors influencing the administration of certain categories, among a large sample of preschool children of the Municipality of Athens, Greece. According to our findings, the vast majority of parents have administered at least one MWP, including antibiotics, to address the symptoms of common childhood diseases. Factors associated with the use of MWP, particularly analgesic-antipyretics and antibiotics, were the child's age, father's education, and parental nationality, as well as knowledge and information regarding NPM, their source of advice, with most representative the pharmacist, and finally their general knowledge and perceptions concerning the purchase and use of the MWP.

As reported by recent data, NPM in children is often used without sufficient indication, thus increasing the risk of adverse reactions [8, 12]. Therefore, international scientific organizations as well as state health agencies, including the World Health Organization and the US Food and Drug Administration (FDA), have mainly emphasized informing parents about the dangers following the administration of those NPM that are most widely consumed and often considered harmless [12–15]. The upward trend in NPM consumption in children, especially those used for the treatment of fever and the common cold, has been well described worldwide [1, 2, 16, 17]. Results from a previous study showed that 98.1% of parents in different countries had used at least one NPM for their children in the 12-month period before the collection of relevant data, a proportion similar to that recorded in the present study [1, 2]. The latter was an interesting finding because until now the supply of NPM in our country was possible only through pharmacies, leading to the assumption that this would possibly result in their reduced consumption.

According to our results, fever was the main symptom for the administration of antipyretics, while acetaminophen was the preferred formulation, followed by mefenamic acid. Children belonging to the age group of 4–6 years were more likely to receive antipyretics-analgesics compared to those of 2–4 years old, possibly due to the increasing confidence of parents with their child's advancing age. Similar studies performed by others report that more than 50% of preschool children have used NPM, most frequently those less than two years of age, with acetaminophen being the medicine of choice for the treatment of fever and pain [1, 2, 16–18]. This difference may be attributed to the fact that children in our country enroll in kindergartens and are consequently exposed to infections, at older ages. The use of mefenamic acid instead of ibuprofen as a second-choice antipyretic may be attributed to the familiarity of this formulation in Greece.

Since May 2011, the Food and Drug Administration Committee, responsible for NPM, issued new guidelines for the administration of acetaminophen removing the indication “pain relief” from the packaging as well as from the package inserts, for both infants and young children, considering that there is no reliable evidence of its effectiveness in relieving pain compared to placebo, especially for children under the age of two years [19]. It is noteworthy that acetaminophen has been described as the most common cause of acute liver failure in the US, which may occur at younger ages even when the dose is within the recommended limits [20]. The above measures are expected to decrease the incidence of adverse events attributed to acetaminophen as well as its overuse, following the example of a cough and cold medication restriction by the FDA in 2008 [21, 22].

This study showed that, among antibiotics delivered to children without a prescription, second generation cephalosporins were most frequently administered for the treatment of upper respiratory tract infections, whereas amoxicillin was administered for common cold symptoms. It is well known that infections of the upper respiratory tract are the most common cause of antibiotic use in everyday pediatric practice even though their majority are of viral origin and do not require antimicrobial treatment [23]. According to reports by the European Agency of Surveillance of Antibiotic Consumption (ESAC), Greece is the European country with the most extensive use of antibiotics in the community, which appears to have further increased by 25% during the economic crisis [11, 23, 24]. More specifically, cefuroxime use has been recorded to have an upward trend as a treatment option for infections of the respiratory tract [11, 24]. Interestingly, in a survey conducted in Greece in 2014, among 60% of respondents that received information concerning antibiotic consumption by media, only 1 in 5 was aware that these were prescription-only medicines. Unfortunately, this malpractice is ongoing despite the fact that antimicrobials are classified by law, since 1976, as “strictly prescription-only medicines” [11] and despite the implementation of a nationwide electronic drug prescription system during the last few years. Part of the problem is the ease with which pharmacists provide these medicines, as has been recorded in previous studies conducted in our country [25, 26]. In our study, the incidence of children using antibiotics without prescription was lower, compared to another similar study concerning adults where the respective use of was 18.6% [11]. Even though we found that antibiotics were administered at a significantly lower rate than other medicines, their average use was more extensive than that of dermatological or cough preparations. Specifically, the majority of parents marked all seven different groups of trade names of antibiotics that were provided in the questionnaire, namely, more than the particular formulations of the other drug categories, indicating their widespread use in the community.

The lower educational level and foreign nationality appeared to increase the possibility of administration of antipyretics and antibiotics significantly. This may be associated with the socioeconomic status of parents and their difficulty towards access to health services. Easy access to these medicines and lower socioeconomic status have been recorded to facilitate their use in the past, particularly in the lack of pediatric advice [27]. This study contrasts data from other countries reporting increased NPM administration in children among parents of higher socioeconomic and educational level, mostly attributed to convenience issues and their lack of time for visiting a physician [1, 2, 28].

Prompt consultation from a pediatrician reduced the likelihood of using antipyretics, whereas advice by relatives had the opposite effect, indicating their strong influence on the family's health issues in our country. Information obtained through the Internet and television did not appear to have a significant influence on the administration of antipyretics-analgesics, unlike findings from an Italian study [29]. Finally, parents who disagreed that medicines should only be administered after a pediatric consultation were significantly more likely to use unprescribed antibiotics, while the probability of using both antipyretics and antibiotics increased significantly when information was sought by a pharmacist.

To our knowledge, this is the first study providing insight into the outpatient consumption of MWP, including both NPM and POM as well as information on factors potentially affecting their administration among a large cohort of preschool children. The low response rate (43.1%) is a limitation of the study, as nonparticipating parents might present worse attitudes regarding MWP use. Another limitation of our study was that, since it was conducted in the Municipality of Athens among families of low and middle socioeconomic level, our findings cannot be generalized. Nevertheless, they may provide useful information on attitudes and associated factors that drive parents to use NPM. Finally, our results highlight the ongoing local malpractice of POM supply through pharmacies without the necessary prescription, stressing the need for continuous education, and stricter intervention policies in our country.

## 5. Conclusion

We found that an alarming proportion of parents in our country have administered medication without a prescription, including antibiotics, to their preschool children. Several sociodemographic factors, as well as the role of certain categories of health professionals, were identified as predictors for the above use, highlighting targets for intervention. The implementation of information campaigns at a national level, targeting parents and health professionals, regarding the prudent use of medication without a prescription in children and the serious consequences of their improper use as well as the strict control of disposing prescription-only medicines are considered essential.

## Figures and Tables

**Table 1:** Nonprescribed medicines: administration of antipyretics-analgesics (AA) according to child's symptoms.

Active substance	Fever *N*, (%)^*∗*^	Fever & pain *N*, (%)^*∗*^	Cold *N*, (%)^*∗*^	High fever *N*, (%)^*∗*^	Localized pain *N*, (%)^*∗*^	Pain *N*, (%)^*∗*^	Cough *N*, (%)^*∗*^	Total *N*, (%)^*∗∗*^
*Acetaminophen*	1255 (57.1)	160 (66.1)	88 (63.8)	42 (40.8)	27 (61.4)	7 (77.8)	2 (66.7)	1581 (57.8)
*Mefenamic acid*	773 (35.2)	60 (24.8)	42 (30.4)	45 (43.7)	12 (27.3)	1 (11.1)	1 (33.3)	934 (34.1)
*Tolfenamic acid*	96 (4.4)	11 (4.6)	4 (3.0)	7 (6.8)	0 (0.0)	0 (0.0)	0 (0.0)	118 (4.3)
*Ibuprofen*	70 (3.2)	10 (4.1)	3 (2.2)	9 (8.7)	4 (9.1)	1 (11.1)	0 (0.0)	97 (3.6)
*Acetylsalicylic acid*	3 (0.2)	1 (0.4)	1 (0.7)	0 (0.0)	1 (2.3)	0 (0.0)	0 (0.0)	6 (0.2)

Total *N*1 (%) of medicines	2197 (100.0)	242 (100.0)	138 (100.0)	103 (100.0)	44 (100.0)	9 (100.0)	3 (100.0)	2736 (100.0)
Total *N*2 (%) of parents	1379 (78.0)^*∗∗∗*^	180 (10.2)	92 (5.2)	68 (3.9)	39 (2.2)	8 (0.5)	2 (0.1)	1768 (100.0)

*N*1: number (%) of AA medicines administered in total for each symptom. *N*2: number (%) of parents administering AA for each symptom. ^*∗*^Number (%) of each AA formulation administered for each symptom over the total number of AA given for the same symptom. ^*∗∗*^Number (%) of each AA formulation administered for all symptoms over the total number of medicines. ^*∗∗∗*^The number (%) of parents administering AA for fever over the total number of parents using AA for all symptoms.

**Table 2:** Prescription-only medication: administration of antibiotics according to child's symptoms.

Active substance	Upper respiratory tract infection	Cold	Fever & cough	Lower respiratory tract infection	Localized pain^*∗*^	Other infections	Total
	*N*, (%)^*∗∗*^	*N*, (%)^*∗∗*^	*N*, (%)^*∗∗*^	*N*, (%)^*∗∗*^	*N*, (%)^*∗∗*^	*N*, (%)^*∗∗*^	*N*, (%)^*∗∗∗*^
*Amoxicillin*	34 (23.6)	24 (41.4)	16 (39.0)	6 (16.2)	5 (27.8)	1 (14.3)	86 (28.2)
*Cefaclor*	19 (13.2)	3 (5.2)	3 (7.3)	1 (2.7)	2 (11.1)	2 (28.6)	30 (9.8)
*Cefprozil*	15 (10.4)	2 (3.5)	1 (2.4)	0 (0.0)	2 (11.1)	2 (28.6)	22 (7.2)
*Cefuroxime*	10 (6.5)	2 (3.5)	1 (2.4)	4 (10.8)	1 (5.6)	0 (0.0)	18 (5.9)
*Amoxicillin/clavulanic acid*	42 (29.2)	14 (24.1)	9 (22.0)	11 (29.7)	6 (33.3)	1 (14.3)	83 (27.2)
*Clarithromycin*	20 (13.9)	12 (20.7)	10 (24.4)	12 (32.4)	2 (11.1)	1 (14.3)	57 (18.7)
*Azithromycin*	4 (2.8)	1 (1.7)	1 (2.4)	3 (8.1)	0 (0.0)	0 (0.0)	9 (3.0)

Total *N*1 (%) of medicines	144 (100.0)	58 (100.0)	41 (100.0)	37 (100.0)	18 (100.0)	7 (1.0)	305 (100.0)
Total *N*2 (%) of parents	110 (45.5)	45 (18.6)	35 (14.5)	29 (12.0)	16 (6.6)	7 (2.9)	242 (100.0)

*N*1: number of antibiotic formulations administered in total for each symptom. *N*2: number of parents who administered antibiotics for each symptom. ^*∗*^Ear pain, sore throat, and toothache. ^*∗∗*^Number (%) of antibiotics administered for each symptom over the total number administered for the same symptom. ^*∗∗∗*^Number (%) of antibiotic formulations administered for all symptoms over the total number of antibiotics used.

**Table 3:** Multivariable model to explore factors associated with the administration of antipyretics-analgesics.

Factors	OR^*∗∗*^	(95% C.I)^*∗∗∗*^	*p* value
*Child's age group in years*			0.011
≤2	0.64	(0.242–1.684)	0.364
>2–4	0.61	(0.416–0.902)	0.013
>4–6^*∗*^	1		
≥6	0.07	(0.012–0.427)	0.004
No answer	1.21	(0.245–6.021)	0.813
*Father's educational level*			0.018
Compulsory	0.6	(0.351–1.016)	0.057
High school graduate^*∗*^	1		
University graduate	0.69	(0.440–1.071)	0.097
Postgraduate	0.36	(0.192–0.684)	0.002
No answer	1.07	(0.348–3.306)	0.903
*Mother's nationality*			0.038
Greek^*∗*^	1		
Others	1.75	(1.030–2.983)	0.038
Marital status			0.04
Married^*∗*^	1		
Single	0.79	(0.365–1.729)	0.561
Divorced	0.36	(0.160–0.795)	0.012
*Are you familiar with the term “medicines given without prescription” *			<0.001
No	0.27	(0.139–0.503)	<0.001
Yes^*∗*^	1		
No answer	3.27	(1.808–5.905)	<0.001
*Time elapsed before requesting paediatric consultation*			<0.001
<1 day	0.38	(0.237–0.610)	<0.001
1-2 days^*∗*^	1		
2–4 days	1.8	(0.818–3.981)	0.144
>4 days	0.91	(0.303–2.744)	0.87
I did not ask for consultation	0.4	(0.188–0.831)	0.014
No answer	0.24	(0.114–0.512)	<0.001
*Did you first refer to the pharmacy box in your house?*			0.048
No	0.83	(0.530–1.315)	0.435
Yes^*∗*^	1		
No answer	0.35	(0.154–0.809)	0.014
*Were you certain that you had all relevant information?*			0.032
No	0.49	(0.287–0.836)	0.009
Yes^*∗*^	1		
No answer	1.02	(0.382–2.730)	0.967
*Were you aware of the active substances and ingredients included in the medicines you administered? *			0.011
No	0.97	(0.611–1.545)	0.903
Yes^*∗*^	1		
No answer	0.25	(0.102–0.624)	0.003
*I tend to buy medicines that are advertised *			<0.001
No^*∗*^	1		
Yes	0.35	(0.134–0.907)	0.031
No answer	103.09	(11.920–891.554)	<0.001
*I tend to buy medicines that friends or relatives have used*			0.004
No^*∗*^	1		
Yes	0.59	(0.208–1.690)	0.328
No answer	0.04	(0.005–0.274)	0.001
*Were you informed by several sources regarding the medication? *			0.03
No	0.47	(0.236–0.930)	0.03
Yes^*∗*^	1		
*Were you informed by the pharmacist?*			0.017
No^*∗*^	1		
Yes	1.77	(1.106–2.815)	0.017
*Were you informed by relatives?*			0.03
No^*∗*^	1		
Yes	7.47	(1.216–45.926)	0.03
*Were you informed by a TV-advertisement?*			<0.001
No^*∗*^	1		
Yes	0.04	(0.007–0.188)	<0.001
*Were you informed by the Internet?*			0.016
No^*∗*^	1		
Yes	0.38	(0.169–0.836)	0.016

^*∗*^Reference category; ^*∗∗*^OR = Odds Ratio; ^*∗∗∗*^95% CI = 95% Confidence Interval.

**Table 4:** Multivariable model for the exploration of factors associated with the administration of antibiotics.

Factor	O.R^*∗∗*^	(95% C.I.)^*∗∗∗*^	*p* value
*Parental age*	0.97	(0.946–1.002)	0.067
*Gender of parent completing the questionnaire*			0.032
Male	1.53	(1.098–2.137)	0.012
Female^*∗*^	1		
No answer	3.61	(0.239–54.550)	0.354
*Father's nationality*			<0.001
Greek^*∗*^	1		
Other	2.22	(1.619–3.041)	<0.001
No answer	2.62	(1.270–5.414)	0.009
*Time elapsed before requesting pediatric consultation *			0.054
<1 day	1.04	(0.751–1.451)	0.799
1-2 days^*∗*^	1		
2–4 days	1.05	(0.715–1.527)	0.822
>4 days	1.65	(0.908–3.006)	0.1
I did not ask for consultation	0.43	(0.213–0.882)	0.021
No answer	0.59	(0.262–1.318)	0.197
*Did you try to remember how you acted in the past in a similar situation regarding another of your children?*			0.095
No	0.97	(0.722–1.304)	0.84
Yes^*∗*^	1		
No answer	0.58	(0.349–0.956)	0.033
*The residual medicine you:*			<0.001
Threw away	2.49	(1.851–3.344)	<0.001
Kept for future use^*∗*^	1		
No answer	1.35	(0.693–2.628)	0.378
*I buy medicines prescribed by my pediatrician in the past that I know are suitable for this particular health issue *			0.005
I disagree	0.61	(0.454–0.827)	0.001
I agree^*∗*^	1		
No answer	1.01	(0.421–2.424)	0.982
*I buy medicines that are popular by name *			0.001
I disagree	0.6	(0.451–0.807)	0.001
I agree^*∗*^	1		
No answer	1.37	(0.664–2.845)	0.392
*I consult my pediatrician before administering any medication*			<0.001
I disagree	2.81	(1.981–3.981)	<0.001
I agree^*∗*^	1		
No answer	2.62	(1.053–6.519)	0.038
*I am able to tell whether my child's health issue is not serious and can therefore wait without using any treatment*			0.022
I disagree^*∗*^	1		
I agree	0.77	(0.583–1.027)	0.076
No answer	2.61	(0.977–6.993)	0.056
*The difficulty of finding an open pharmacy discourages me from using medicines*			0.002
I disagree^*∗*^	1		
I agree	1.45	(1.077–1.939)	0.014
No answer	0.33	(0.125–0.854)	0.022
*Were you informed by the pharmacist?*			0.054
No^*∗*^	1		
Yes	1.31	(0.996–1.724)	0.054

^*∗*^Reference category; ^*∗∗*^OR = Odds Ratio; ^*∗∗∗*^95% CI = 95% Confidence Interval.
